# The Intimate Connection between Areas of Low Barometer and the Prevalence of Epidemics of Non-Contagious Diseases

**Published:** 1887-01

**Authors:** T. C. Osborn

**Affiliations:** Cleburne, Texas


DANIEL'S
### Texas Medical Journal.

PUBLISHED MONTHLY.—SUBSCRIPTION $2.00 A YEAR.

Vol. 2. AUSTIN, JANUARY, 1887. No. 7.

### ORIGINAL ARTICLES.

CONTRIBUTED EXCLUSIVELY TO THIS JOURNAL.

*The Articles in this Department are accepted and published with the understanding that we are not responsible for, nor do we indorse the views and opinions of the writers by so doing.*

### THE INTIMATE CONNECTION BETWEEN AREAS OF LOW BAROMETER, AND THE PREVALENCE OF EPIDEMICS OF NON-CONTAGIOUS DISEASES.

*By T. C. Osborn, M. D., Cleburne, Texas.*

For Daniel's Texas Medical Journal.

I FIND it very difficult to frame an apology for failing, according to my promise to furnish an article for the December issue of your JOURNAL, for on attempting the task, I was confronted with the all important fact that other meteorological influences were, perhaps, quite as necessary in the work as the barometer, thermometer, wind-vane, and rain-guage, which were the only instruments I possessed for making weather observations. And now, at this late day, I am constrained by these circumstances, to appeal to your generous forbearance, and to ask you to accept at present a letter as introductory to the subject, which, if my life is spared, I may have a greater abundance of evidence for substantiating the opinions that are herein announced. In the performance of that task, I crave the liberty of asking your influence with the profession in Texas for corroborative assistance, to the end that the work may be accomplished satisfactorily to science, and to the State in which the discovery emanated. The cost of a corrected Green's thermometer, a sensitive aneroid barometer, rain-guage, and wind-vane, is so trifling that the most indigent physician in the State can bear it without injury to himself and family, and the pleasure of recording tri-daily observations on these instruments will amply compensate him for the investment. This duty leads one very soon to anticipate the changes of the weather, and teaches the intelligent physician when it is best to weaken or strengthen the vital forces of his patients, aside from the indications presented by the system, in the diseases which he is employed to relieve; especially if he is at the same time, as no doubt all are doing, making a conscientious record in a book for that purpose, of each case coming under his observation for treatment. But if there should unfortunately be one amongst them so shamefully derelict in his obligations to the profession, and sluggishly negligent of the imperative duties he owes to his clientelle, I hope he will permit me to assure him that the pleasure he will derive from keeping such records as these, will repay him a thousand-fold for the care and trouble he has taken in the work.

The main object of this letter, therefore, is simply to *announce* the inferences I have drawn from a four years meteorological and medical sources of observation in this area, and to solicit physicians everywhere in the State to unite with me in tabulating,with this view,similar observations for monthly comparison at a central station; and I know of no better station for such a grand work than in DANIEL'S TEXAS MEDICAL JOURNAL, provided the editor will kindly consent to its insertion, and supervise and arrange the tabulation in the best manner to attract the attention of the readers of his pages.

As far as the meteorological observations are concerned, I have no reason to doubt the willingness of the Signal Service Department to furnish "blank forms" to physicians in every neighborhood, provided it may receive monthly summaries in return for such favors; and with these forms the trouble of making observations will be reduced to its minimum quantity. But the Department will not furnish instruments to voluntary observers. This stinginess on the part of the Commissioner is, doubtless, due to the "penny wise and pound foolish" opposition of Congress in appropriations for this grandest science in the whole domain of scientific literature; and is the more to be deplored by suffering humanity, if the announcement I am making should prove to be a true hint in the right direction.

It is not possible, with so few instruments as I possess, to arrive at a satisfactory solution of the question of the direct relationship between diseases, and certain conditions of the weather; but there are high probabilities in my favor of appreciating the approach to the solution, when I see that a similarity in the movements of the atmosphere in this area, are constantly followed by a tendency to definite displays of epidemic diseases amongst the people with whom I am associated. This is quite as true as my ability, through a series of faithful observations, to refute the foul slander so often repeated that "no one can tell any thing of the weather in Texas." In truth, there is as much regularity in the weather in this State, as in any other, if I may take barometrical evidence for granted. As atmospheric areas are very numerous, and very often of limited extent in circumference, there is but little that is wonderful in the slander; and I feel quite confident that the day is not distant when the laws of the atmospheric currents which we breathe, will be as well understood as the surface upon which we stand. But before that day arrives, and to hasten its coming, it is not only necessary, but highly important that the physicians of Texas should arm themselves—at least one in each neighborhood—with meteorological instruments, and work concertedly upon the problem that, at present, seems so mysteriously abstruse. Each, and all of them, are fully cognizant of the influence of weather disturbances upon human health and longevity, and each and every one of them ought to be held responsible by their clientelle for criminal neglect of professional duty, who fail to inform themselves of the laws of such disturbances, so as to be able to instruct others in the best method of protecting those over whose health they are the chosen custodians.

Nor is this all. The strong hand of the law should be laid with no gentle pressure upon that physician who is derelict in keeping a faithful record-book, in which is conscientiously stated the clinical history, and every thing else connected with the disease of which he is at the time writing, not merely for his own use, but as well for the noble profession of which he is a member. He well knows that medical facts accumulate only by logical deduction, the force of which is to be strengthened or weakened by subsequent experience.

If the foregoing postulates are granted, no physician should be permitted to practice medicine who is too ignorant or too lazy, to observe the weather, or to trust only to memory as a record of the diseases he has daily to meet. Yet how often are seen physicians in the court-room, who make affirmations to details before a jury from memory alone, even where life itself is, perhaps, at stake!

But now, after such an apologetic episode, I hope you will still bear with me in my attempt to disclose "the evidence of the faith that is within me." Neither is it probable, I trust, that you will tire over the humdrum statistics which, believe me, are essentially necessary to a full explanation of the position adopted in this discussion.

The city of Cleburne, the county seat of Johnson, has a population of upwards of 5,000 souls; is 790 feet above the gulf level, and is located upon lands lying between, and just above the junction of East and West Buffalo creeks. Geographically it is situated in latitude 32° 25 m. north and longitude 97° 18 m. west. On the northeast, east, southeast, and south for many miles in extent, the soil is made up of coarse sand, the land is undulating and heavily covered with timber; whilst the northwest, west, and southwest is an extensive undulating prairie land, barren of timber as far as the sight can reach, only along the various streams, amongst which are the Nolan and Brazos rivers, where, in many places, the forest is abundant enough to supply fire-wood for many years to the dense population in their immediate vicinities. All the streams, and there are quite a number of them, each trending towards the south, have gravelly beds, and the fall is so great that freshets can last but a few hours, leaving lagging pools sometimes a mile or more in length, in which the water supply is abundant enough for stock purposes even during fearful droughts, because they are fed by numerous and apparently inexhaustible springs.

This picture has a healthy outlook, and according to the county statistics, it is so in reality.

During my residence here for the last four years, I have been industriously occupied in medical and meteorological observations, but owing to much imperfection in the weather tables for 1883, I am unwilling to include that year in the series, only so far as the statement is concerned in medicine, in which was observed an unusual number of cases of a septicæmic character; and very many cases of congestion of the kidneys. These conditions were apparent to every observer; so much so, that our urinometers and microscopes were held in almost constant service. I am very sure that I saw more cases of kidney troubles in that year alone, than in all my experience before.

Septic complications were remarkably abundant in all cases of lying-in rooms, and in surgical wards it was rare to find them entirely absent. But whether these various troubles were due to atmospheric low pressure, I have not the evidence in my imperfectly kept record of that year to determine. It is certain, however, that the same seasons in which they occurred, our city and the surrounding country had an unusual number of cases of malarial fevers, hardly any place escaping the infliction; which goes a great way towards a conviction that bacterial life was very active, and there can be but little doubt of the prolonged existence of low barometer, not only in this area, but also in the counties immediately surrounding us. It is now an admitted proposition that, bacilli are most prolific, as well as most destructive in their habits, during a low condition of the atmosphere. We are better able in this way to account for the continual prevalence of the yellow fever of the South, because, at and near the equator,there is a much lower pressure of the atmosphere than at the tropics and the poles. In fact, the yellow fever declines in violence as it approaches tropical regions, and unless the contagion meets with a favorable pabulum in the form of atmospheric low pressure, it rarely progresses beyond the 30th degree of north latitude, even without a shadow of quarantine surveilance to obstruct its march. Whereas, on the other hand, whenever the barometer steadily indicates a pressure below a normal reading, the infection flies through the most rigid quarantines, in spite of the best directed efforts to prevent it. How long, think you, would the contagion of yellow fever live, under one of our brisk northern blasts, such as we frequently have in Texas? The "northers," as we call them, are precisely the opposites of low barometer, and constitute one of the greatest blessings an all wise Creator confers upon human existence. If we value long and healthy life, for this blessing we ought never cease expressing our grateful recognition; for without their reanimating influences, longevity could have no possible existence except in imagination. For a short time during each norther, the diathermacy of the atmosphere is almost as perfect as it is on the highest American altitudes, and the few mouthfuls of pure. cold, fresh air is more acceptable to the nervous and vascular systems of men and animals, and richer in healthful ingredients, than that of all the other hygienic possibilities combined. High barometer in this sense, means high life to the healthy, and renewed hope to the consumptive invalid. The reason is plain. Microphytic life in atmospheric high pressure is then at its minimum capacity, and bacillary energy is in a great measure arrested by the inervation due to the rarity and purity of the atmosphere.

This mode of reasoning correctly explains the tabulated statistics of the mortality from phthisis pulmonalis, showing the least number of deaths in the late autumn season, and greatest percentages in the middle spring months. These sad fatalities of the spring season, are directly dependent upon the low barometric pressure of that season, more then than any other in the year, and, as a matter of course, there are more enervating influences at work upon the already enfeebled system, than during the seasons of atmospheric high barometer, such as I have stated in the beginning of this paragraph.

Low barometer means, in my view of it, asthma, erysipelas, neuralgia, diphtheria, pneumonia. rheumatism, meningitis, and many other afflictions; besides being a boquet to the medical man within its area.

In the latter part of February, 1884, my barometer fell to 28.90, and the temperature at the same time was 60°. On the page of the record of that day, (18th) I mention, "cloudy morning, fine afternoon; lightning northest at 8 a. m.; sunset in bank of strati; cirri abundant at intervals all day; thunder in the south at 11 p. m." This quotation is prefatory to the comments to be made on the subsequent spring months, and is important as a pointer to the kind of weather which might well be anticipated. The winter had been remarkably severe, and serious damage was observed, in many places, to the grain crops.

For March the mean barometer was 29.28; thermometer 57°; rainfall 6¼ inches; frost on the 2d and 9th; prevailing winds south, southeast, southwest; and 8 thunder storms during the month.

April, mean barometer 29.23; thermometer 60°; rainfall $3\frac{64}{100}$

inches; frost 9th and 23d; prevailing winds northwest, north, northeast; and 10 thunder storms.

May, mean barometer. 29.26; thermometer, 68°; rainfall, $5\frac{29}{100}$ inches; winds northwest, north, northeast, south, southeast, southwest; and 13 thunder storms.

June, mean barometer, 29.27; thermometer, 75°; rainfall $10\frac{91}{100}$ inches; winds southeast, south, southwest; and 11 thunder storms.

July,mean barometer, 29.22; thermometer, 84; rainfall $0\frac{32}{100}$ inches; winds southeast, south, southwest; and 2 thunder storms.

August,mean barometer, 29.31; (this, I think, is in the neighborhood of the normal reading) thermometer, 85°; rainfall $0\frac{62}{100}$ inches; winds south, southeast, southwest; and 4 thunder storms.

September, mean barometer, 29.27; thermometer, 81°; rainfall $\frac{102}{100}$ inches; winds south, southeast, southwest; and 1 thunder storm.

The total means for the seven months are: barometer, 29.27; thermometer, 72°; rainfall $28\frac{09}{100}$ inches (an excess for this area); and 49 thunder storms.

Beginning with March we had an epidemic of such diseases as rheumatism, meningitis, pneumonia, and later in the season, say in June, after these had subsided, there were an unusual number of cases of eczema, with quite a variety of other dermal afflictions; and after the middle of August, many mild cases of typho-malarial fevers.

In 1885. the means for the seven months, from the first of March to the first of October, were as follows: Barometer, 29.33; thermometer, 71°; and 35 thunder storms. This year was characterized as free from epidemic diseases, with the exception of a sprinkling of dengue in September, during which month the mean barometer declined to 22.29. But this was of short duration, as on the 4th of October we had the first norther of the season, and the barometer reached 29.49, or about 15 points above the mean normal reading. During the summer months there were several cases of typho-malarial fever, but hardly any other regular form of disease worth noticing.

In 1886, the mean barometer for the seven months from March to September inclusive was 29.29, or several points below normal; whilst the thermometer mean for the same time was 71°; rainfall $15\frac{73}{100}$; and 26 thunder storms.

In April began an epidemic of rotheln, which lasted until June, and was quickly followed by epidemic dengue, that continued broadcast over the city until the 13th of the following November, at which time we had our first killing frost; the thermometer stood at 24°, and the barometer ran up to 29.69. The record reads as follows: "Clear sunrise, killing frost, ice, calm; 1 horizon strati southeast to southwest; cirro-strati at 2 p. m.; cloudy sunset, and inappreciable rainfall at 8 p. m."

Of the non-contagious epidemic of rotheln, which, with very rare exceptions. was confined to children, there were four deaths, due mainly to imprudent exposure to drafts of night air. Deaths from this mild form of the order of exanthemata, is exceedingly rare, and I believe, never occurs unless from the cause stated above. Having during my professional career observed several epidemics of rotheln, or German measles; rosalina, or Italian measles; or roseola, English measles, as this disease is variously designated, I am not at all disposed to agree with the authorities as to its contagious nature. On the contrary, it has seemed to me as truly atmospheric in its origin, as it was non-contagious in character. It is generally confined to single areas, appearing in a number of different families at the same time, with perhaps, rarely more than two cases, and very frequently only one, leaving no marks of infection behind it, even where there were a number of subjects in the same house.

But, like diphtheria, which is contagious, *if we may rely* upon the testimony of respectable physicians, in Europe and the northern portions of the United States, whilst in this latitude (32° N.,) where I have been often associated with epidemics of it for nearly half a century, there is not the least evidence to me of its infectious nature. Often enough, I have known only one out of a numerous family to take the disease, and even when more than one had it, they were afflicted almost simultaneously, with results strongly similar; that is, if there was a first case fatal, the others were quite sure to prove fatal, until several deaths occurred in a short time in the family. I have known mothers, and two or three of their children, to be stricken with diphtheria at nearly the same time, without the remotest probability of connection between these cases, and those of other families in the same vicinity. I cannot, therefore, from my individual experience, agree in the opinion that diphtheria is a contagious disease in this latitude. The true explanation is, I think, to be found in the theory of barometric curves, and especially within areas of low barometer. Why not? No one can doubt the influence of atmospheric low pressure upon the health of men, as well as animals. And if the doubt should exist in the minds of physicians, that doubt would quickly disappear when the epidemic met its match with the appearance of an opposite atmosphere of high pressure, by seeing the epidemic arrested at once in its course. The same thing can be safely asserted as to the epidemics of rotheln, dengue, erysipelas, pneumonia, meningitis, neuralgia and asthma. They are all caused by atmospheric low pressure, as certainly as they are modified, or arrested by an opposite condition of the atmosphere.

It is my custom to say to patients afflicted with pneumonia who are breathing with difficulty, especially if my barometer warns me of an approaching norther, "be patient a few hours longer, and you will get a mouthful of pure, fresh, cold air, which will make you feel decidedly better"; and I am invariably assured at the next visit of the correctness of the prediction. Early in my practice, I was in the habit of surrounding pneumonia patients with a heavy bed covering, and stopping off every current of cold air from the room. But the case is now very different. I would a thousand times rather treat the disease out doors than in the stagnant air of a close room.

Of late years it is unpleasant to my feelings to differ from the standard medical authorities, but surely, the same right of thinking for myself on the subjects of rotheln and dengue, especially as they seem to claim the same privileges in reference to their classification and contagious character. And it seems to me that modesty would dictate the assertion that I am open to conviction, whenever the proof is amply sufficient that my position is wrong, is presented to my consideration. But, even whilst saying this, I feel confident that such evidence will not be offered me in the few remaining years of my life. My opportunities have been ample enough during forty-six years of professional life to warrant me in my convictions of the non-infectious nature of both these diseases, and especially as my observations during several epidemics have been mainly directed towards a solution of that question.

In conclusion, I will, in recapitulation, offer to the profession the following propositions, namely :

1st. Atmospheric low pressure is often limited to small areas, and is probably the cause of non-contagious diseases assuming an epidemic character within those areas.

2d. It is more in the number of such diseases than in the contagiousness, that the appearance of infections exists, as it is difficult at times to distinguish the difference, owing to the apparently close intimacy existing between the frequently recurring cases in the same vicinity.

3d. That whatever form an epidemic may assume, it is quickly brought to an end by an opposite condition of the atmosphere, or high pressure of the barometer, if continued but a few days. and is accompanied with strong currents of cold air.

4th. Dengue strictly belongs to Good's class iii, Hæmatica; Order iii exanthematica; and Genus i enanthesis, or rash exanthem. It is preceded by a fever for a definite time until the eruption appears, and this in turn is followed by desquamation; fever and discomfort subsiding with the appearance of efflorescence. It is non-contagious, and as a rule, recurs with every epidemic, or, if preferred, creates no immunity to subsequent invasions. And, judging from my observations, it requires besides low barometer, less rainfall, or humidity, and a diminished electrical condition of the atmosphere, for the production of the epidemic.

5th. That rotheln epidemics, as well as a variety of other forms of epidemic skin diseases, non-contagious in character, are developed only by atmospheric conditions similar to dengue, and like it, may recur in the same area, and in the same iudividuals more than once, under precisely the same atmospheric condition.

And now, my dear Doctor, having laid down my predicates, and touched gently upon what I believe to be a keynote to a vast field of useful information, I will turn the paper over to you, with the fervent hope that you will consent to assist me in arousing a deep interest in the profession in every portion of the State; so that, before a decade has passed, we shall have an abundance of evidence to rely upon as to the truth or falsity of my theory.

